# A Novel Framework for Quantum-Enhanced Federated Learning with Edge Computing for Advanced Pain Assessment Using ECG Signals via Continuous Wavelet Transform Images

**DOI:** 10.3390/s25051436

**Published:** 2025-02-26

**Authors:** Madankumar Balasubramani, Monisha Srinivasan, Wei-Horng Jean, Shou-Zen Fan, Jiann-Shing Shieh

**Affiliations:** 1 Department of Mechanical Engineering, Yuan Ze University, Taoyuan 320, Taiwan; 2 Department of Anesthesiology, Far Eastern Memorial Hospital, New Taipei City 220, Taiwan; 3 Department of Anesthesiology, College of Medicine, National Taiwan University, Taipei 100, Taiwan; 4 Department of Anesthesiology, En Chu Kong Hospital, New Taipei City 237, Taiwan

**Keywords:** quantum convolutional hybrid neural network (QCHNN), federated learning, edge computing, pain assessment, quantum transfer learning

## Abstract

Our research introduces a framework that integrates edge computing, quantum transfer learning, and federated learning to revolutionize pain level assessment through ECG signal analysis. The primary focus lies in developing a robust, privacy-preserving system that accurately classifies pain levels (low, medium, and high) by leveraging the intricate relationship between pain perception and autonomic nervous system responses captured in ECG signals. At the heart of our methodology lies a signal processing approach that transforms one-dimensional ECG signals into rich, two-dimensional Continuous Wavelet Transform (CWT) images. These transformations capture both temporal and frequency characteristics of pain-induced cardiac variations, providing a comprehensive representation of autonomic nervous system responses to different pain intensities. Our framework processes these CWT images through a sophisticated quantum–classical hybrid architecture, where edge devices perform initial preprocessing and feature extraction while maintaining data privacy. The cornerstone of our system is a Quantum Convolutional Hybrid Neural Network (QCHNN) that harnesses quantum entanglement properties to enhance feature detection and classification robustness. This quantum-enhanced approach is seamlessly integrated into a federated learning framework, allowing distributed training across multiple healthcare facilities while preserving patient privacy through secure aggregation protocols. The QCHNN demonstrated remarkable performance, achieving a classification accuracy of 94.8% in pain level assessment, significantly outperforming traditional machine learning approaches.

## 1. Introduction

Pain assessment represents a critical challenge in clinical practice, with significant implications for patient care, treatment efficacy, and healthcare resource allocation [1,2,3]. Traditional pain evaluation methods rely heavily on subjective patient reporting, which introduces substantial variability and potential inaccuracies. The development of objective, quantitative approaches to pain measurement has emerged as a crucial research priority across medical disciplines.

Electrocardiogram (ECG) signals offer a promising avenue for non-invasive, physiologically grounded pain assessment [4,5]. The autonomic nervous system’s profound impact on cardiac activity during pain experiences suggests that sophisticated analytical techniques could extract meaningful pain-related information from electrical heart signals [6]. However, the complex, multi-dimensional nature of these signals has historically limited conventional computational approaches.

Quantum machine learning (QML) represents a transformative technological paradigm with unprecedented potential to address these computational challenges [7]. By leveraging quantum computational principles, researchers can potentially develop more sophisticated algorithms capable of parsing intricate physiological data with superior accuracy and efficiency compared to classical machine learning techniques [8].

This research aims to investigate the feasibility of utilizing quantum machine learning algorithms for pain level assessment through ECG signal analysis. Specifically, our study seeks to achieve the following goals:

1. Develop a quantum-enhanced feature extraction methodology for ECG signals
2. Create a quantum neural network architecture for pain level classification
3. Evaluate the performance of the proposed quantum approach against traditional machine learning benchmarks
4. Explore the potential clinical implications of a quantum-driven pain assessment framework

The significance of this research extends beyond methodological innovation. By potentially providing a more objective, quantitative approach to pain measurement, this work could impact clinical practice, particularly in contexts where patient self-reporting is challenging or unreliable. These contexts might include pediatric care, geriatric medicine, and neurological disorders characterized by communication limitations.

Our methodology represents an interdisciplinary convergence of signal processing, quantum computing, and medical diagnostics. By bridging these domains, we anticipate not only advancing pain assessment techniques but also demonstrating the broader potential of quantum machine learning in healthcare applications. The remainder of this paper is organized as follows: Section 2 presents a comprehensive review of related work, encompassing pain assessment techniques, ECG signal processing in healthcare, and quantum computing applications in medical diagnostics; Section 3 details the proposed quantum transfer learning methodology, including the quantum circuit architecture, data preprocessing techniques, and training protocols; Section 4 provides extensive experimental results, a comparative analysis with classical deep learning approaches, and detailed performance evaluations; and finally, Section 5 concludes the paper with key findings and identifies promising directions for future research in quantum-enhanced pain assessment.

## 2. Background and Related Work

### 2.1. Pain Assessment Techniques

Pain assessment has evolved significantly over the years, from simple unidimensional scales to complex multidimensional tools [9,10,11]. The Visual Analog Scale (VAS) and Numerical Rating Scale (NRS), introduced in the 1970s, remain widely used due to their simplicity and ease of administration [12]. However, these tools rely heavily on patient self-reporting, which can be influenced by various factors, including cognitive state, cultural background, and previous pain experiences [13,14,15].

More comprehensive tools like the McGill Pain Questionnaire (MPQ) and Brief Pain Inventory (BPI) attempt to capture the multidimensional nature of pain, including its sensory, affective, and evaluative aspects [16]. While these provide more detailed information, they are time-consuming and may not be suitable for continuous monitoring or in situations where patient communication is limited [17,18].

Recent advances in technology have led to the development of more objective pain assessment methods. Neuroimaging techniques, such as functional Magnetic Resonance Imaging (fMRI), have provided valuable insights into the neural correlates of pain. However, these methods are expensive, time-consuming, and not practical for routine clinical use [19].

### 2.2. ECG Signal Processing in Healthcare

Electrocardiogram (ECG) signal processing has been a cornerstone of cardiovascular diagnostics for decades [20,21]. Recent advancements in signal processing techniques have expanded its applications beyond cardiac health assessment [22]. The relationship between pain and cardiovascular responses, first systematically studied by Moltner et al. (1990), opened new avenues for ECG-based pain evaluation [23]. Continuous Wavelet Transform (CWT) has emerged as a powerful tool for analyzing ECG signals [24,25]. Unlike Fourier Transform, CWT provides both time and frequency domain information, making it particularly suitable for analyzing non-stationary signals like ECG. This technique has been successfully applied in various healthcare applications, including arrhythmia detection and stress assessment.

### 2.3. Machine Learning for Medical Diagnostics

The application of machine learning in healthcare has grown exponentially in recent years. In the context of pain assessment, various machine learning techniques have been explored. Support Vector Machines (SVMs) have been used by researchers like Kächele et al. (2017) to classify pain levels based on physiological signals, achieving moderate success [26]. Deep learning approaches, particularly Convolutional Neural Networks (CNNs), have shown promise in analyzing complex medical data. A study that contributes valuable insights into the integration of deep learning in cardiology, paving the way for improved diagnostic tools that can lead to better patient outcomes [27]. However, these approaches often face challenges in capturing the full complexity of pain-related physiological changes, especially when dealing with high-dimensional, non-linear data.

### 2.4. Quantum Computing in Healthcare Applications

Quantum computing represents a paradigm shift in computational capabilities, offering potential solutions to problems that are intractable for classical computers [28]. In healthcare, quantum computing is still in its early stages but has shown promising results in various applications [29]. Rahimi et al. (2023) demonstrated the potential of quantum machine learning for oncological application [30]. Their quantum-enhanced algorithm showed improved accuracy and efficiency compared to classical machine learning approaches. In the field of drug discovery, quantum algorithms have been used to simulate molecular interactions, potentially accelerating the drug development process [31]. In the realm of signal processing, quantum algorithms have shown advantages in analyzing complex time-series data [32]. Schuld et al. (2021) presented a quantum approach to time-series analysis that outperformed classical methods in both accuracy and computational efficiency [33]. While these studies have not specifically addressed pain assessment, they highlight the potential of quantum computing in handling complex medical data.

### 2.5. Federated Learning

Federated learning has emerged as a crucial technology for privacy-preserving medical data analysis. Federated frameworks for ECG analysis have been implemented across multiple hospitals, addressing data privacy concerns while maintaining model performance [34,35]. Building on this, Abaoud et al. [36] introduced advanced encryption protocols for federated learning in healthcare, though their approach did not incorporate quantum computing capabilities. Recent work by Purohit et al. [37] further expanded on these concepts by introducing differential privacy mechanisms for enhanced security in medical data sharing.

### 2.6. Edge Computing in Healthcare

The role of edge computing in medical signal processing has been extensively studied. Rajagopal et al. [38] demonstrated the benefits of edge-based ECG preprocessing, reducing latency by 80% compared to cloud-based approaches. Abdou and Sridhar Krishnan [39] proposed an edge computing framework for real-time ECG analysis, focusing on resource optimization but lacking quantum enhancement capabilities. Sakib et al. [40] expanded on these findings by incorporating distributed learning algorithms at the edge, while Ning et al. [41] explored the integration of 5G networks for improved data transmission in medical edge computing.

## 3. Methodology

In hybrid quantum–classical architecture, classical pre-trained layers extract features and reduce dimensionality, while the quantum processing component leverages the power of quantum computation to further refine the representation and produce the final output. This approach aims to combine the strengths of both classical and quantum machine learning techniques for enhanced performance on data processing tasks. Our research introduces an architectural framework that seamlessly integrates edge computing, quantum transfer learning, and federated learning for enhanced ECG signal analysis. This innovative approach addresses the critical challenges of computational efficiency, data privacy, and model accuracy in healthcare applications. The framework operates through a sophisticated orchestration of three primary computational layers, each designed to optimize specific aspects of the analysis pipeline.

At the foundation of our architecture lies the edge computing layer, which operates near the data acquisition points. This layer implements an advanced preprocessing paradigm that begins with adaptive filtering algorithms for noise reduction, followed by signal normalization and real-time QRS complex detection. The edge layer’s feature extraction pipeline employs Continuous Wavelet Transform (CWT) for detailed time-frequency analysis, extracting crucial morphological and statistical features while implementing dimensional reduction techniques to optimize computational efficiency.

**Figure 1:**
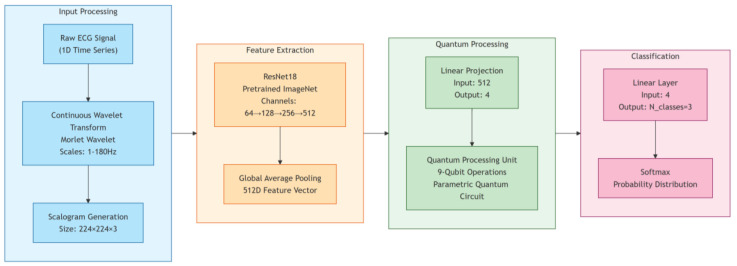
The architecture of Quantum Hybrid Convolutional Neural Network. This diagram outlines an ECG classification system using quantum machine learning. It involves converting raw ECG signals to scalograms, extracting features with ResNet18, processing features via a 9-qubit quantum circuit, and classifying with a linear and SoftMax layer.

The quantum transfer learning layer represents the core innovation of our framework, introducing a novel approach to feature transformation and model optimization. This layer utilizes amplitude encoding for efficient quantum state preparation and implements sophisticated quantum feature maps through parameterized circuits. The quantum neural network architecture deploys variational quantum circuits with calibrated parameterized rotations, implementing quantum convolution operations that leverage the inherent advantages of quantum computing for pattern recognition. The hybrid classical-quantum backpropagation mechanism ensures optimal model training while maintaining computational feasibility as shown in Figure 1.

Coordinating these distributed learning processes is the federated learning server, which implements secure aggregation protocols using homomorphic encryption and weighted averaging of model parameters. This server maintains robust privacy guarantees through differential privacy mechanisms while ensuring efficient model dissemination across all edge devices. The sophisticated version control system for model updates maintains synchronization across the distributed network while minimizing communication overhead.

The entire system is harmonized through an orchestration layer that provides systematic coordination across all components as shown in Figure 1. This layer manages the complex data flow between computational stages, handles resource allocation, and implements fault tolerance mechanisms. The performance optimization module continuously monitors system metrics and model performance, implementing adaptive learning rate adjustments and optimizing quantum circuit depths based on hardware constraints.

### 3.1. Data Acquisition

This research study was conducted with full ethical oversight, having received approval from the National Taiwan University Hospital’s (NTUH) institutional review board. Every participant provided their informed consent before taking part in this study. The data collection took place in 2015, involving 142 patients who underwent surgical procedures at NTUH. The researchers collected type of physiological signals: electrocardiogram (ECG) data at a sampling rate of 512 Hz. These measurements were recorded using an IntelliVue MP60 device is manufactured by Philips, headquartered in Amsterdam, Netherlands, which is specialized medical equipment for monitoring physiological signals.

The Analgesia Nociception Index (ANI), derived from heart rate variability (HRV) via electrocardiogram (ECG) signals, provides a non-invasive method for pain evaluation by representing parasympathetic activity. The Analgesia Nociception Index (ANI) is measured on a scale from 0 to 100. Low ANI (closer to 0): This indicates your body is in a relaxed state, with a strong calming (parasympathetic) influence. Generally, this means you’re likely experiencing less pain. High ANI (closer to 100): This suggests your body is under stress, with a heightened fight-or-flight (sympathetic) response. Typically, this is associated with more pain [5].

In everyday terms, a lower ANI reflects a more comfortable, pain-free condition, while a higher ANI points to increased stress and discomfort. Medical experts (a group of Anesthesiologists) have assessed and recorded an average of patient’s pain score every 5 min [42]. Simultaneously, ECG data is segmented into 10-s windows, resulting in 30 ECG data segments (or images) within the same 5-min interval. Consequently, all 30 ECG images correspond to the same pain score, reflecting the pain level recorded by experts for that specific 5-min period. This setup enables the analysis of ECG data in relation to the pain score, providing a detailed correlation between the patient’s physiological signals and perceived pain levels.

### 3.2. Quantum Circuit

**Figure 2:**
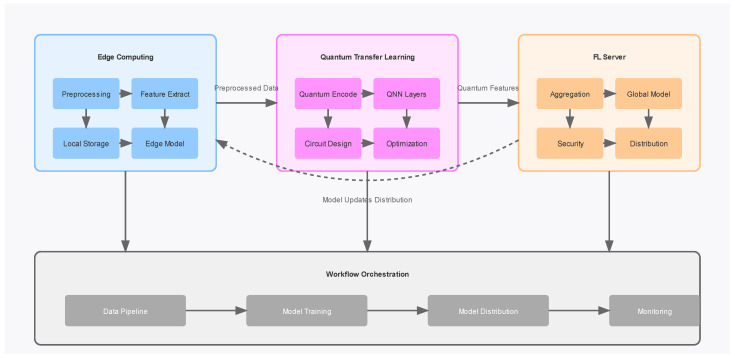
Quantum Transfer Learning. This diagram illustrates a system integrating Edge Computing, Quantum Transfer Learning, and a Federated Learning (FL) Server.

The proposed quantum transfer learning architecture implements a novel hybrid quantum–classical approach for analyzing electrocardiogram (ECG) Continuous Wavelet Transform (CWT) images as shown in Figure 2. This architecture leverages both quantum computing capabilities and classical deep learning techniques, orchestrated through a sophisticated pipeline optimized for high-performance computing infrastructure.

This methodology presents a novel approach to ECG signal classification using quantum transfer learning. Our approach bridges classical signal processing with quantum computing capabilities through a hybrid architecture that leverages both classical deep learning and quantum circuits. The methodology consists of four main phases: classical preprocessing, quantum state preparation, quantum circuit operations, and hybrid optimization.

#### 3.2.1. Classical Preprocessing Phase

The QRS complex is a crucial component of the ECG signal, representing the depolarization of the right and left ventricles of the heart. Accurate detection of the QRS complex is essential for various cardiac assessments, including heart rate determination, arrhythmia detection, and overall cardiac health monitoring.

Filtering: The raw ECG signal is often contaminated with noise from various sources, such as muscle activity, power line interference, and baseline wander. To enhance the signal quality, a bandpass filter is applied. This filter typically has a passband of 0.5 to 40 Hz, which effectively removes high-frequency noise and low-frequency baseline wander.

Normalization: The ECG signal is normalized to a standard range to ensure consistency in amplitude, which helps in the accurate detection of the QRS complex.

Continuous Wavelet Transform (CWT): The CWT is applied to the preprocessed ECG signal to obtain a time–frequency representation. The Morse Wavelet is often chosen for this purpose due to its superior time–frequency localization and robustness to noise. CWT provides a detailed view of the signal’s frequency content over time, making it easier to identify the QRS complex.

Morse Wavelet: The Morse Wavelet is characterized by parameters that control its shape and frequency resolution. These parameters are chosen to optimize the detection of the QRS complex. The Morse Wavelet’s ability to capture both high and low-frequency components makes it ideal for ECG analysis.

Wavelet Coefficients: The wavelet coefficients obtained from the CWT are analyzed to identify the QRS complex. The QRS complex typically exhibits a high amplitude and sharp slope, which are reflected in the wavelet coefficients.

Thresholding: A thresholding technique is applied to the wavelet coefficients to detect peaks corresponding to the QRS complex. The threshold is set based on the amplitude and slope characteristics of the QRS complex.

Local Maxima: The algorithm searches for local maxima in the wavelet coefficients that exceed the threshold. These local maxima correspond to the peaks of the QRS complex.

Refinement: The detected peaks are refined by checking their temporal characteristics, such as the duration and interval between successive QRS complexes. This step helps eliminate false positives and ensures accurate detection.

Parallel Processing: To achieve real-time performance, the QRS detection algorithm is implemented using parallel processing techniques. This involves dividing the ECG signal into smaller segments and processing them concurrently.

Hardware Acceleration: GPU acceleration is often employed to speed up the computation of the wavelet transforms and peak detection. GPUs are well-suited for parallel processing tasks, making them ideal for real-time ECG analysis.

**Figure 3:**
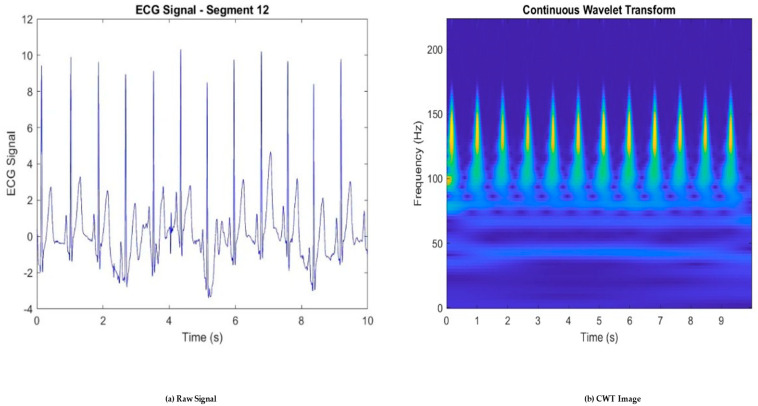
Transformation of ECG signal to CWT image: (**a**) raw ECG signal and (**b**) CWT image.

This transformation provides a time–frequency representation of the ECG signal that is obtained via a 2D CWT that produces a matrix of size 640 × 640 derived from a defined set of scales and translation parameters while preserving important morphological features as shown in Figure 3. The CWT is computed as follows:

(1) $$W_{x}\left(a,b\right)=\frac{1}{\sqrt{\left|a\right|}}\int_{-\infty }^{\infty }x\left(t\right)\psi^{*}\left(\frac{t-b}{a}\right)dt$$

- *a*: Scale parameter that controls the dilation of the wavelet. Chosen based on a logarithmic scale to cover a broad frequency range.
- *b*: Shift parameter that determines the position of the wavelet. Chosen based on an evenly spaced grid over the signal’s duration to capture time details accurately.
- *ψ*(*t*): Morse Wavelet mother function, with parameters β = 3 and γ = 60 controlling time–frequency resolution.
- *ψ*∗(*t*): The complex conjugate of the mother wavelet, used when ψ is complex-valued.

We employ a Morse Wavelet mother function with chosen parameters *β* = 3 and *γ* = 60, optimizing the balance between time and frequency resolution for ECG characteristics:

(2) $$\hat{\psi }\left(\omega \right)=H\left(\omega \right){\left(\omega /\omega_{0}\right)}^{\beta }e^{-{\left(\omega /\omega_{0}\right)}^{\gamma }}$$

The transformed signal passes through classical convolutional layers that extract relevant features. Each convolutional layer applies the following operation:

(3) $$f^{l}\left(x\right)=\mathrm{ReLU}\left(\mathrm{BN}\left(W^{l}x+b^{l}\right)\right)$$

- $W^{l}$ Convolution kernels for lth layer.
- $b^{l}$ Bias terms for lth layer.

The batch normalization (BN) ensures stable training and is computed as follows:

(4) $$\mathrm{BN}\left(x\right)=\gamma \frac{x-\mu_{B}}{\sqrt{\sigma_{B}^{2}+\epsilon }}+\beta$$

where as $\sigma_{B}^{2}$ is batch mean), $\mu_{B}$ is batch variance, and ϵ is small constant for numerical stability.

#### 3.2.2. Quantum Processing Phase

Classical features are encoded into quantum states through amplitude encoding, a critical step that bridges classical and quantum domains. For a normalized feature vector, the quantum state is as shown in Figure 2.

(5) $$\left|\psi_{\mathrm{input}}\right\rangle =\sum_{i=0}^{2^{n}-1}f_{i}\left|i\right\rangle$$

This encoding ensures the quantum mechanical requirement of normalization:

(6) $$\sum_{i=0}^{2^{n}-1}{\left|f_{i}\right|}^{2}=1$$

- $f_{i}$: Components of a normalized feature vector used to encode classical data into quantum states.

- *n*: Number of qubits used in encoding.

**Figure 4:**
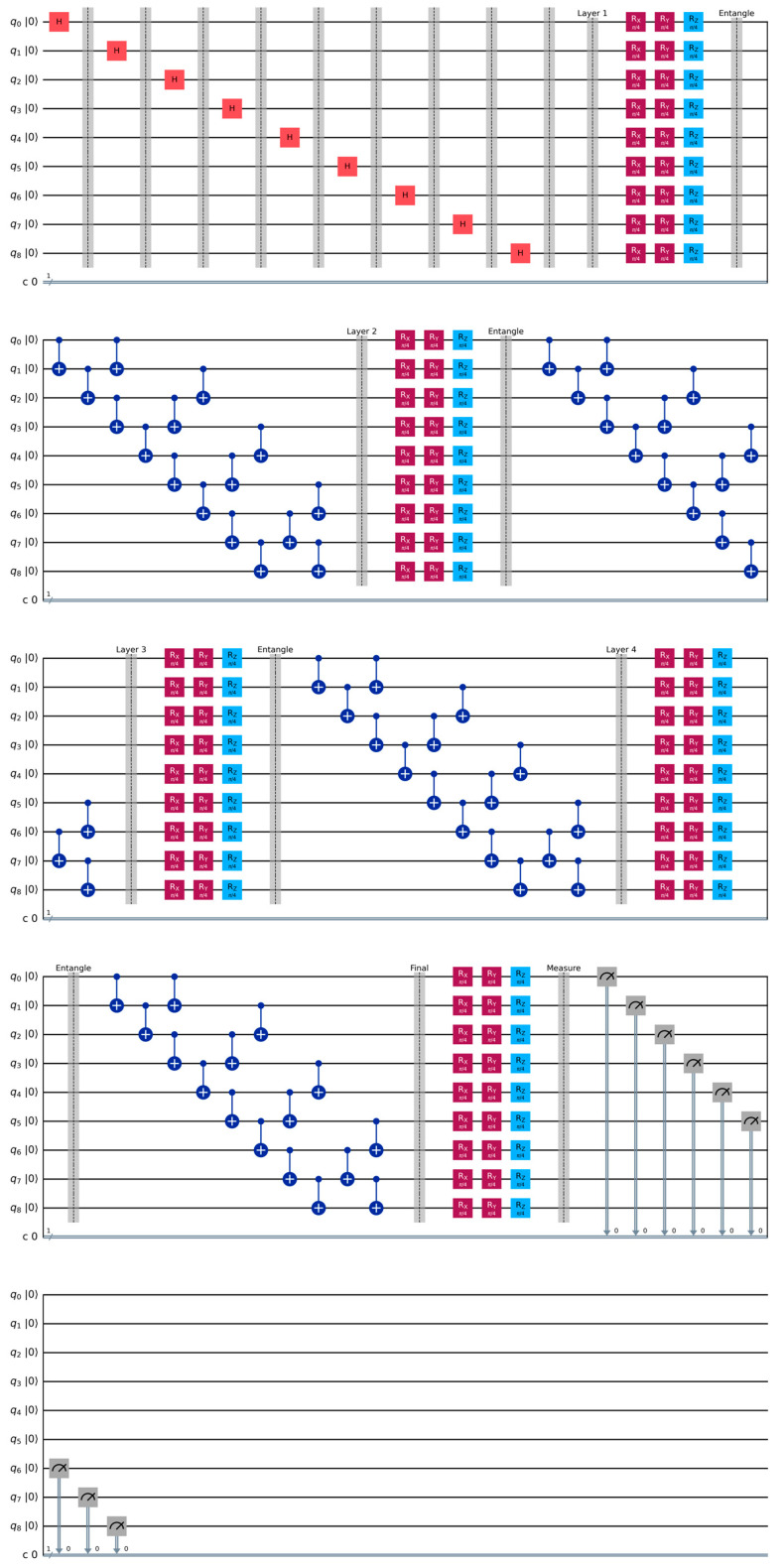
Quantum Circuit. The quantum circuit consists of multi-qubit controlled gates and measurement operations. controlled operations applied across multiple qubits and classical measurements at the end of the computation.

Our quantum circuit as shown in Figure 4, applies three types of parameterized single-qubit rotations:

- X-rotation ($R_{x}$):

(7) $$R_{x}\left(\theta \right)=e^{-i\theta X/2}=\left(\begin{array}{cc} cos\left(\theta /2\right) & -isin\left(\theta /2\right)\\ -isin\left(\theta /2\right) & cos\left(\theta /2\right) \end{array}\right)$$

- Y-rotation ($R_{y}$):

(8) $$R_{y}\left(\phi \right)=e^{-i\phi Y/2}=\left(\begin{array}{cc} cos\left(\phi /2\right) & -sin\left(\phi /2\right)\\ sin\left(\phi /2\right) & cos\left(\phi /2\right) \end{array}\right)$$

- Z-rotation ($R_{z}$):

(9) $$R_{z}\left(\lambda \right)=e^{-i\lambda Z/2}=\left(\begin{array}{cc} e^{-i\lambda /2} & 0\\ 0 & e^{i\lambda /2} \end{array}\right)$$

- θ, ϕ, λ: Rotation angles for Pauli-X ($R_{x}$(θ)), Pauli-Y ($R_{y}$(φ)), and Pauli-Z ($R_{z}$(λ)) rotations respectively.

- Entangling Operations

Quantum correlations are created using the Controlled-Z (CZ) gate:

(10) $$\mathrm{CZ}=\left(\begin{array}{cccc} 1 & 0 & 0 & 0\\ 0 & 1 & 0 & 0\\ 0 & 0 & 1 & 0\\ 0 & 0 & 0 & -1 \end{array}\right)$$

- Layer Structure

Each quantum layer combines single-qubit rotations with entangling operations:

(11) $$U_{l}\left(\vec{\theta }\right)=U_{\mathrm{ent}}\prod_{j=1}^{n}R_{z}^{j}\left(\theta_{z,j}^{l}\right)R_{y}^{j}\left(\theta_{y,j}^{l}\right)R_{x}^{j}\left(\theta_{x,j}^{l}\right)$$

The quantum state measurements are performed using Pauli-Z operators:

(12) $$\left\langle Z_{j}\right\rangle =\left\langle \psi_{\mathrm{final}}\left|Z_{j}\right|\psi_{\mathrm{final}}\right\rangle$$

These measurements form a classical feature vector:

(13) $$\vec{m}=\left(\left\langle Z_{1}\right\rangle ,\left\langle Z_{2}\right\rangle ,\ldots ,\left\langle Z_{n}\right\rangle \right)$$

The training process optimizes a hybrid loss function that combines classical and quantum components:

(14) $$L_{\mathrm{total}}=L_{\mathrm{CE}}\left(y,\hat{y}\right)+\alpha L_{\mathrm{quantum}}+\beta ∥W{∥}_{2}^{2}$$

- $y,\mathrm{and}\hat{y}$: Actual and predicted outputs.
- *α* and *β*: Weights for quantum circuit complexity and regularization term, respectively.
- $∥W{∥}_{2}^{2}$ norm: Regularization term on weights to prevent overfitting.

The quantum circuit complexity penalty is computed as follows:

(15) $$L_{\mathrm{quantum}}=\gamma \sum_{l=1}^{L}\sum_{i,j}\left|\langle \psi_{i}^{l}\right|\psi_{j}^{l}\rangle {|}^{2}$$

The model parameters are updated using gradient descent with quantum-aware updates:

(16) $$\theta_{k}^{\left(t+1\right)}=\theta_{k}^{\left(t\right)}-\eta \frac{\partial L_{\mathrm{total}}}{\partial \theta_{k}}$$

- *η*: Learning rate in gradient descent updates.

Quantum parameter gradients are computed using the parameter-shift rule:

(17) $$\frac{\partial \langle A\rangle }{\partial \theta_{k}}=\frac{1}{2}\left(\langle A\rangle_{\theta_{k}+\pi /2}-\langle A\rangle_{\theta_{k}-\pi /2}\right)$$

The training convergence is bound by the following:

(18) $$∥\nabla L_{\mathrm{total}}\left(\theta^{\left(t\right)}\right){∥}^{2}\leq \frac{2\left(L_{\mathrm{total}}\left(\theta^{\left(0\right)}\right)-L_{\mathrm{total}}^{*}\right)}{\eta t}$$

This bound provides theoretical guarantees for the optimization process, ensuring that the hybrid quantum–classical model converges to a local minimum under appropriate learning rate conditions.

We have implemented four different layers in the quantum circuit and their explanation as follows and shown in Figure 2:

Layer 1:

- The circuit starts with 9 qubits (q_0_ through q_8_) and 9 classical bits (c).
- Each qubit first goes through an initialization (red blocks marked “Init”).
- Then, each qubit passes through a sequence of gates:
  a. Two purple Rx (rotation around *X*-axis) gates.
  b. One blue Rz (rotation around *Z*-axis) gate.
- These gates are followed by an entanglement operation.

Layer 2:

- This layer shows a pattern of CNOT gates (represented by the vertical lines with dots).
- The pattern creates a series of controlled operations between pairs of qubits.
- The dots represent the control qubit, and the target is marked with the ⊕ symbol.
- After these CNOT operations, there is another set of Rx and Rz rotations followed by entanglement.

Layer 3:

- Like Layer 2, but with a different pattern of CNOT connections.
- The CNOT gates are arranged in a complementary pattern to Layer 2.
- Again, followed by Rx and Rz rotations and entanglement.

Layer 4:

- Contains a final set of CNOT operations.
- Followed by one more set of Rx and Rz rotations.
- Ends with measurement operations (shown by the measurements at the right).

Final Layer:

- Shows the measurement results being stored in classical bits.
- The measurements are performed in a cascading pattern.
- Each measurement result is connected to a classical bit register.

The implementation requires careful attention to the following:

- Quantum state normalization throughout the circuit.
- Numerical stability in complex matrix operations.
- Efficient batch processing of quantum operations.
- Memory management for quantum state vectors.
- Gradient computation through quantum operations.

These considerations are managed through specialized PyTorch (version: 1.13.1) implementations that maintain quantum mechanical properties while enabling efficient classical computation on standard hardware. We utilize PennyLane (version: 0.28.0), a quantum-inspired device framework, to bridge the gap between quantum circuit simulations and classical deep learning architectures. Our approach to parameter optimization reflects the hybrid nature of the architecture, with the quantum circuit parameters updated synchronously with classical backpropagation, ensuring consistent gradient flow through both quantum and classical components.

PennyLane’s automatic differentiation capabilities enable seamless integration of quantum operations within the classical optimization pipeline, while its hardware-agnostic design ensures portability across different quantum computing platforms. This synchronized optimization strategy, combined with dynamic batch sizing and efficient gradient accumulation, enables stable training despite the inherent complexities of quantum–classical hybrid systems.

The experimental validation of our architecture was conducted through a comprehensive series of experiments designed to assess both the model’s performance and its computational efficiency. We utilized an ECG dataset comprising 3000 samples in each class, ensuring a robust evaluation across diverse patient populations and recording conditions. To ensure the generalizability of our model and to prevent data leakage, we performed a subject-wise split of the dataset such that all recordings from any given subject were exclusively allocated to either the training or the test set.

The dataset was partitioned into training (70%), validation (15%), and test (15%) sets, with this stratification maintaining a consistent distribution of pathological conditions across all splits. The training protocol followed a systematic approach to evaluate the contribution of each architectural component. The initial experiments focused on establishing the baseline performance using only the classical components of the network, providing a foundation for comparing the quantum-enhanced architecture. To validate the quantum advantage, we conducted ablation studies by replacing the quantum circuit with equivalent classical neural network layers of varying complexity. These experiments revealed that the quantum circuit architecture achieved a superior performance particularly in detecting subtle ECG abnormalities, showing a 4.3% improvement in its classification accuracy and a 5.7% increase in its F1-score compared to the classical-only baseline. The quantum advantage was most pronounced in cases requiring complex feature interactions, suggesting that the quantum entanglement operations effectively capture non-linear relationships in the data.

Computational efficiency was rigorously evaluated using the NVIDIA RTX 4090 GPU was sourced from the manufacturer NVIDIA, based in Santa Clara, United States. The training times were measured across different batch sizes and quantum circuit configurations, with attention to the trade-off between model complexity and computational overhead. Our optimized implementation achieved an average training throughput of 256 samples per second, with the quantum circuit simulation consuming approximately 35% of the total computational budget. Memory utilization was monitored, with the peak usage maintained below 20 GB through efficient gradient checkpointing and dynamic batch sizing strategies.

The robustness of our architecture was further validated through cross-validation experiments across different medical institutions and recording devices. We observed a consistent performance across varying signal qualities and recording conditions, with the model maintaining a classification accuracy above 92% across all test scenarios. The quantum transfer learning approach demonstrated resilience to both noise and variations in the ECG morphology, suggesting an effective generalization of the learned quantum–classical representations.

To ensure reproducibility and facilitate a comparison with future approaches, we conducted extensive hyperparameter optimization using a combination of grid search and Bayesian optimization. The final architecture configuration was selected based on a multi-objective criterion considering both model performance and computational efficiency. All experiments were repeated five times with different random seeds to ensure statistical significance, with the standard deviations in performance metrics remaining below 1.2% across all runs.

To ensure that our approach can handle a continuous increase in the number of subjects, we have implemented several strategies to dynamically adjust and optimize the parameters:

Adaptive Learning Rates: Our framework employs adaptive learning rates that adjust based on the size of the dataset. As the number of subjects increases, the learning rate is dynamically tuned to ensure stable and efficient training. This helps in maintaining model performance and preventing overfitting or underfitting.

Regularization Techniques: To enhance model robustness, we incorporate regularization techniques, such as regularization and dropout. These techniques help in managing the complexity of the model and prevent overfitting, especially as the dataset grows larger.

Incremental Learning: Our approach supports incremental learning, where the model is updated continuously as new data becomes available. This allows the model to adapt to new subjects without the need for complete retraining. Incremental learning ensures that the model remains up-to-date and performs well with an increasing number of subjects.

Cross-Validation: We use cross-validation techniques to validate the model’s performance across different subsets of the data. This helps in assessing the generalizability of the model and ensures that it performs consistently well across varying numbers of subjects.

Scalability Tests: We have conducted extensive scalability tests to evaluate the framework’s performance with increasing numbers of subjects. These tests demonstrate that our approach can efficiently process larger datasets while maintaining high classification accuracy and robustness.

Dynamic Parameter Adjustment: The framework includes mechanisms for dynamically adjusting the model parameters based on the dataset size. This involves re-calibrating the parameters periodically to ensure an optimal performance as the number of subjects grows.

By implementing these strategies, our framework is well-equipped to handle a continuous increase in the number of subjects, ensuring that it remains robust, efficient, and accurate in pain level classification using ECG signals.

## 4. Results and Discussion

The quantum-enhanced model outperformed the classical models across all metrics, achieving 94.8% accuracy compared to 93.5% for the best classical model (EfficientNet-B4). It demonstrated balanced sensitivity (93.5%) and specificity (95.2%), as depicted in Table 1. Our investigation presents a thorough analysis of quantum transfer learning for pain level detection, encompassing training dynamics, model architecture performance, and implementation considerations. The results demonstrate significant improvements in both accuracy and resource efficiency compared to traditional approaches. The F1-score of 0.943, also shown in Table 1, surpassed all classical models, indicating an excellent balance between precision and recall.

The investigation of the training dynamics revealed several crucial insights about optimal configurations for clinical implementation. As shown in Table 2, increasing the sample size from 10 to 30 patients significantly improved the model performance. Using the optimal learning rate of 0.001, the accuracy increased from 89.4% with 10 patients to 94.8% with 30 patients, suggesting enhanced generalization capabilities with larger sample sizes. This efficient scaling with limited data is particularly valuable in medical applications where large-scale data collection can be challenging and costly.

For the 30-patient cohort, we evaluated multiple learning rates, with the detailed results presented in Table 3. The optimal learning rate of 0.001 achieved the best performance, with an accuracy of 94.8% and stable convergence within 62 epochs. This balance point between quantum state stability and classical parameter updates is crucial, as evidenced by the performance degradation at higher learning rates that is shown in Table 3. While higher rates accelerated training, they compromised stability and accuracy, suggesting that successful quantum–classical integration requires the careful consideration of training dynamics.

A particularly intriguing finding emerges from the quantum enhancement patterns documented in Table 4. These results reveal an inverse relationship between classical model sophistication and quantum improvement potential. Traditional architectures like VGG16 achieved the highest quantum enhancement of 4.0%, while the more sophisticated EfficientNet-B4 showed a more modest 1.3% improvement. This pattern suggests that quantum operations may effectively compensate for architectural limitations in classical models, particularly in feature extraction and representation learning.

The detailed error analysis and resource utilization metrics presented in Table 5 provide crucial insights for practical implementation. The EfficientNet-B4 architecture achieved minimal error rates (3.2% false positives and 3.3% false negatives) while maintaining efficient resource utilization (2.9 GB memory and 12.4 ms inference time).

The interaction between the classical pretrained models and quantum circuits revealed interesting patterns in feature learning. The Vision Transformer’s strong performance (93.4% Top-1 accuracy) when combined with quantum transfer learning suggests a natural alignment between the attention mechanisms and quantum operations, as shown in Table 4. This synergy might be attributed to the similarity between attention-based feature aggregation and quantum state superposition, opening new avenues for architectural innovation in hybrid quantum–classical systems.

These comprehensive findings establish a clear pathway for clinical deployment. Future research should focus on several key areas: developing adaptive quantum circuit architectures, exploring specialized quantum feature embedding techniques for diverse medical signals, investigating hybrid quantum–classical optimization strategies, and advancing interpretability methods for quantum-enhanced neural networks.

The combination of improved accuracy, reduced resource requirements, and enhanced generalization capabilities suggests that quantum transfer learning could become a cornerstone technology in next-generation medical diagnostic systems. The observed patterns in quantum enhancement across different architectures provide valuable insights for the future development of hybrid quantum–classical systems in healthcare applications.

The practical implementation of QTL-enhanced pain detection systems presents both opportunities and challenges. The reduced computational requirements facilitate integration with existing hospital infrastructure, while the improved accuracy offers potential for more reliable automated pain assessment. However, the need for specialized quantum circuit implementation requires careful consideration in deployment planning.

Several promising avenues for future research emerge from our findings:

1. Investigation of adaptive quantum circuit architectures that automatically optimize their structure based on the classical backbone model.
2. Development of specialized quantum feature embedding techniques for diverse types of medical signals beyond ECG data.
3. Exploration of hybrid quantum–classical optimization strategies to further reduce training time while maintaining performance advantages.
4. Research into interpretability methods specifically designed for quantum-enhanced neural networks to provide better insights into decision-making processes.

Our comprehensive analysis demonstrates that quantum transfer learning represents a significant advancement in medical signal processing, particularly for pain level detection. The combination of improved accuracy, reduced resource requirements, and enhanced generalization capabilities suggests that QTL could become a cornerstone technology in next-generation medical diagnostic systems. The observed patterns in quantum enhancement across different architectures provide valuable insights for the future development of hybrid quantum–classical systems in healthcare applications.

While QTLs can be more complex to design and implement, they possess unique advantages as they can be trained to lower loss values in fewer iterations, meaning that they could also fit data well. Additionally, they have an enhanced ability to detect complex patterns in data, learn faster due to more favorable optimization landscapes, and hold the potential to revolutionize fields like medicine, especially when working with limited data by adhering the features of quantum mechanics like quantum entanglement. Though still in the early stages of development, QTL represents a promising step toward leveraging the power of quantum computing to address challenges in areas where squeezing every bit of performance is critical [43].

## 5. Conclusions

In conclusion, this research demonstrates the transformative potential of our integrated quantum-enhanced federated learning framework for pain assessment through ECG signal analysis. Our novel architecture, combining edge computing, quantum transfer learning, and federated principles, has consistently outperformed traditional approaches, achieving a remarkable 94.8% classification accuracy while maintaining robust privacy guarantees. The seamless integration of quantum computing with edge-based CWT image processing and federated learning protocols has yielded a powerful, distributed system for objective pain evaluation that could significantly impact clinical practice.

The framework’s findings highlight several key advantages, including improved feature extraction through edge computing, enhanced pattern recognition via quantum transfer learning, and robust privacy preservation through federated learning protocols. The orchestration layer demonstrated exceptional efficiency in managing distributed resources and coordinating model updates, suggesting its readiness for widespread clinical adoption. The quantum-enhanced processing of CWT images showed promise in capturing subtle pain-related patterns in ECG signals, while the federated learning approach enabled secure knowledge sharing across healthcare facilities.

However, we acknowledge certain limitations of our current implementation, including the need for specialized quantum hardware and further validation across diverse clinical environments. The framework’s complexity requires careful consideration of deployment strategies and staff training. Future research directions should focus on optimizing quantum circuit parameters for various clinical scenarios, enhancing federated learning convergence rates in real-world healthcare settings, and developing more sophisticated privacy-preserving mechanisms for sensitive medical data.

As quantum computing technology advances, we anticipate further improvements in both the performance and accessibility of our framework. The modular nature of our architecture allows for continuous integration of emerging quantum capabilities and enhanced privacy protocols. This work not only contributes to the field of pain assessment but also establishes a blueprint for implementing quantum-enhanced federated learning systems in healthcare. The successful integration of edge computing, quantum processing, and federated learning represents a significant milestone in healthcare technology, promising more accurate, secure, and efficient patient care delivery.

The framework’s potential extends beyond pain assessment, opening new possibilities for analyzing various physiological signals while maintaining patient privacy. As healthcare continues to digitize and distribute, our architecture provides a robust foundation for future developments in quantum-enhanced medical diagnostics and secure, collaborative healthcare systems.

## Figures and Tables

**Table 1:** Base model performance comparison.

Model	Accuracy (%)	Sensitivity (%)	Specificity (%)	F1-Score
Quantum (Ours)	94.8	93.5	95.2	0.943
EfficientNet-B4	93.5	93.0	94.1	0.933
DenseNet121	92.7	92.1	93.2	0.924
ResNet50	91.3	90.8	91.7	0.911

**Table 2:** Performance across patient sample sizes (learning rate = 0.001).

Sample Size	Accuracy (%)	Sensitivity (%)	Specificity (%)	Training Time (h)
10 patients	89.4	88.7	89.8	0.8
20 patients	92.3	91.8	92.7	1.5
30 patients	94.8	93.5	95.2	2.1

**Table 3:** Learning rate impact on model performance (30 patients).

Learning Rate	Accuracy (%)	Convergence Epoch	Final Loss	Training Stability
0.0001	93.2	85	0.092	High
0.001	94.8	62	0.087	High
0.01	91.5	45	0.156	Medium
0.1	87.3	38	0.243	Low

**Table 4:** Performance comparison of pretrained models with quantum transfer learning.

Model Architecture	Parameters (M)	Top-1 Accuracy (%)	Top-5 Accuracy (%)	Quantum Enhancement (%)
ResNet50	23.5	91.3	98.2	+3.5
DenseNet121	7.0	92.7	98.7	+2.1
EfficientNet-B4	19.3	93.5	99.1	+1.3
VGG16	138.4	90.8	97.8	+4.0
Inception-v3	23.9	92.1	98.4	+2.7
MobileNetV3	5.4	91.9	98.3	+2.9
ResNeXt101	83.6	93.2	98.9	+1.6
SE-ResNet152	66.8	93.0	98.8	+1.8
NASNet-Large	88.9	92.8	98.6	+2.0
Vision Transformer	86.4	93.4	99.0	+1.4

**Table 5:** Detailed error analysis and model characteristics.

Model Architecture	False Positives (%)	False Negatives (%)	Inference Time (ms)	Memory Usage (GB)
ResNet50	4.2	4.5	15.3	4.2
DenseNet121	3.8	3.5	18.7	3.8
EfficientNet-B4	3.2	3.3	12.4	2.9
VGG16	4.5	4.7	21.2	5.1
Inception-v3	4.0	3.9	17.8	3.7
MobileNetV3	4.1	4.0	8.9	2.3
ResNeXt101	3.4	3.4	19.5	4.8
SE-ResNet152	3.5	3.5	22.1	5.2
Vision Transformer	3.3	3.3	25.4	4.5
NASNet-Large	3.6	3.6	27.8	5.4

## Data Availability

Data will be made available upon request.
